# Effect of Health-Promoting Lifestyle Modification Education on Knowledge, Attitude, and Quality of Life of Postmenopausal Women

**DOI:** 10.1155/2020/3572903

**Published:** 2020-05-20

**Authors:** Nirmala Rathnayake, Gayani Alwis, Janaka Lenora, Iresha Mampitiya, Sarath Lekamwasam

**Affiliations:** ^1^Department of Nursing, Faculty of Allied Health Sciences, University of Ruhuna, Sri Lanka; ^2^Department of Anatomy, Faculty of Medicine, University of Ruhuna, Sri Lanka; ^3^Department of Physiology, Faculty of Medicine, University of Ruhuna, Sri Lanka; ^4^Department of Gynecology and Obstetrics, Faculty of Medicine, University of Ruhuna, Sri Lanka; ^5^Population Health Research Centre, Department of Medicine, Faculty of Medicine, University of Ruhuna, Sri Lanka

## Abstract

Limited knowledge and negative attitudes about menopause among postmenopausal women (PMW) create a multitude of health-related issues leading to impaired quality of life (QOL) among them. This study evaluated the impact of a health-promoting lifestyle education intervention (HPLEI) on knowledge, attitude, and QOL in a group of PMW in Sri Lanka. A quasi-experimental study was conducted with 72 PMW, matched for sociodemographic status of the community from two geographically separated areas in Galle, and they were allocated to intervention (*n* = 37) and control (*n* = 35) groups. HPLEI is comprised of health education sessions focused on postmenopausal health management with lifestyle modifications provided only for the intervention group for 8 weeks and follow-up for 6 months. The control group was not given any planned education programme and was allowed to proceed with the usual lifestyle during this period. Knowledge, attitude, menopause-specific QOL (MENQOL), and overall QOL were evaluated in both groups with self-administered questionnaires at the baseline, after 8 weeks of education sessions and at the end of 6 months of follow-up. The mean (SD) ages of the intervention and control groups were 54.6 (4.5) and 56.5 (3.4) (*p* = 0.06) years, respectively. All evaluated variable scores were not different between the intervention and control groups (*p* > 0.05) at the baseline. In the intervention group, knowledge (mean ± SD; 21.70 ± 1.05) and attitude (mean ± SD; 44.02 ± 5.33) scores increased at the end (*p* < 0.001). In the control group, a marginal increase in all dimensions of knowledge scores (mean ± SD; 9.71 ± 2.21) and unchanged attitude scores (mean ± SD; 23.91 ± 7.56) were seen. All MENQOL scores decreased during the follow-up in the intervention group (mean ± SD; 138.51 ± 18.47) (*p* < 0.001) except the sexual domain (*p* = 0.32). MENQOL scores were increased in the control group (mean ± SD; 92.05 ± 28.87) (*p* < 0.001) with time. Overall QOL scores increased (mean ± SD; 74.85 ± 9.71) (*p* < 0.001) in the intervention group during the study period and in the control group overall QOL (mean ± SD; 51.03 ± 13.61) showed a reduction (*p* < 0.001) at the end. Health education focused on health-promoting lifestyle modifications was effective in improving knowledge, attitude, MENQOL, and overall QOL of PMW.

## 1. Introduction

Health education is a primary strategy of health promotion. It is defined as “consciously constructed opportunities for learning involving some form of communication designed to improve health literacy, including improving knowledge, and developing life skills, which are conducive to individual and community health” [1]. Health education is not limited to the dissemination of health-related information and extends to fostering the motivation, skills, and confidence (self-efficacy) that are necessary to take action to improve health [1].

Menopause is a major milestone in women resulting from the depletion of ovarian functions. Menopause leads to a new biological state accompanied by a multitude of physical and psychological changes. It causes a wide range of symptoms such as hot flushes, night sweats, muscle and joint aches or pains, sleeping problems, weight gain, and depression, leading to impairment of quality of life (QOL) [2, 3].

The QOL is defined as “an individual's perception of their position in life in the context of culture and value system in which they live and in relation to their goal expectations, standards and concerns” by the World Health Organization [4]. In postmenopausal women (PMW), QOL usually refers to the aspects pertaining to health based on a combination of symptoms without considering the physical, emotional, or social functions [5]. Therefore, the term QOL specific to PMW is often referred as menopause-specific quality of life (MENQOL) [5].

The women's perception of menopause depends on their social, cultural, and economic status and lifestyle factors. Further, inadequate knowledge and negative attitude towards menopause add to the burden of menopause-related symptoms and impairment of overall QOL at an individual level. This in turn affects the entire family and society, negatively [6, 7].

Women encounter numerous challenges in this period; however, this time is an opportunity for them to change their life and improve their health status. Since some factors are not easily modifiable, lifestyle which is relatively easy to change can be the focus of interventions in this regard. Enhancing the awareness of women regarding physical, psychosocial, and lifestyle changes that arise following menopause through health education programmes is a way to improve positive attitudes towards menopause [8]. Health education with proper training also improves the women's knowledge about menopause enabling them to deal with the emotional and practical aspects of menopause and allow women to be familiar with this stage of life [9]. Furthermore, health standards and QOL can also be enhanced by promoting awareness of changing behaviors and creating an environment that supports good health practices [10] rather than a pharmacologic intervention that has less acceptability proven with greater dropout rates [11]. Therefore, current interventions targeting PMW focus mainly on educating them on disease prevention by adapting a healthy diet and appropriate physical activity schedule [5, 7].

Pender's Health Promotion Model (HPM) is one comprehensive model that emphasizes the promotion of health and the empowerment of individuals for achieving better health and preventing diseases through behavioral changes [12]. This can also be used in postmenopausal health promotion. In Pender's model, behavioral changes are regarded as the desired outcome, and such change is affected by a combination of individual characteristics and experiences, behavior-specific cognitions and attitude, and competing demands and preferences [12]. Existing knowledge, attitude, and irritable menopausal symptoms could be considered as predictors of behavioral change of PMW along with their existing characteristics, experiences, and values. Therefore, a health-promoting activity would direct at attaining positive health outcomes by modifying such factors for aqcuring expected behavioral changes to achive optimal well-being. They will improve health, enhance functional ability, and have better QOL.

Even though previous studies that were conducted guiding HPM [13] have shown positive effects of health education in women around menopause in many countries, no such studies have been reported from Sri Lanka. Further, the application and validity of health education programmes conducted elsewhere to Sri Lankan women is questionable since the experiences of menopause vary across cultures. This should be understood by health care providers, who should strive for women's health [14]. Providing updated, culturally accepted information [15] to PMW with adequate training and supervision can improve their skills, adaptation, and power to accept menopause [16]. Further, if the time allocated for the programme is fairly longer, it would rather enhance its effectiveness and sustainability [15] compared to shorter duration attempts [13]. We assumed that education programmes that focused on updated, culturally accepted information delivered with proper training and supervision for a lengthy period would enhance the QOL of PMW in Sri Lanka effectively.

Improving QOL is imperative for the empowerment of individual PMW for achieving optimum health standards for a country like Sri Lanka since PMW provide valuable contributions as an active labor force and bear many responsibilities in the extended family system. Therefore, the current study based on Pender's HPM was designed to evaluate the impact of a health-promoting lifestyle education intervention (HPLEI) on knowledge, attitude, and QOL in a group of PMW in Sri Lanka.

## 2. Materials and Methods

### 2.1. Study Design, Participants, and Setting

This study was a quasi-experimental study, which observed the impact of HPLEI that was designed as a part of a main study “Effects of menopause on bodily structure, functions and physical health,” conducted at the Faculty of Medicine, University of Ruhuna, Sri Lanka [17]. The methodology on the selection of a sample and HPLEI have been well described in our previous publication when presenting the impact of HPLEI on health-promoting behaviors and health status of the same cohort of study participants [18]. The women (*n* = 166) were selected randomly from 05 Public Health Midwife's (PHM) division in Bope-Poddala Medical Officer of Health area, Galle district, in Sri Lanka, for the main study. During the main study at the initial screening, women on hormone replacement therapy (HRT) or with noncommunicable diseases (NCDs) and disorders related to the musculoskeletal and nervous systems and gait or balance problems were excluded. In addition, women aged 60 years or more or have premature menopause (menopausal age < 40 years), women with menopause secondary to surgery or drug therapy, and women who are exposed to dedicated dietary or exercise programmes currently or previously were also excluded.

Of the 05 PHM divisions, 02 divisions were assigned randomly for the “intervention group (*n* = 42)” and another 02 geographically separated PHM divisions were assigned randomly for the “control group (*n* = 38)” to minimize the contamination. One PHM division was excluded. Consenting women with time since menopause ≥2 to ≤7 years and women who had at least education up to grade 5 were included in the current study.

The sample size calculation was based on a previous study of similar nature done in Iran, by comparing the QOL before and after education intervention [19]. The postmenopausal status was determined on self-stated menstrual history. Only 37 women from the intervention group and 35 women from the control group completed the study (*n* = 72).

### 2.2. Health-Promoting Lifestyle Education Intervention

HPLEI comprised of 8 weeks health education sessions and 6 months of follow-up. Health education included 8 sessions focused on lifestyle modifications which were carried out for 8 weeks (June–July 2017), and printed health education package was provided at the end of the training for the intervention group. A health education package was designed by the research team with the contributions from a group of experts including a gynecologist, physician, nutritionist, and sport physician. It was based on menopausal symptom management, healthy diet [20], healthy physical exercises, and spiritual support, individualized for each participant.

Available options for the management of troublesome menopausal symptoms such as hot flushes and joint pain were provided. Adjustment in diet was done according to the current physical activity level. Proportion of energy was carbohydrate 55–65%, fat 20–30%, and protein 10–15%. The energy distribution of meals was breakfast 20%, lunch 40%, dinner 20%, and snacks 20%. A low-calorie diet (1200-1600 kcal) was recommended concerning the food preferences and available food items. Physical exercises were of three types: continuous walking (30 min ×5 days per week), strength training exercise for limbs (8-10 times ×3 times per day ×3 days per week), and balance training exercise (8-10 times ×3 times per day ×3 days per week). They were also asked to engage in relaxation exercises such as meditation for 10 minutes daily, reading books, listening to music, and engaging in religious activities.

Teaching materials were prepared to suit the subjects including the visual images without medical terminology. The content was culturally acceptable since the content validity was ensured by having a focus group discussion with a group of PMW selected from another geographical location. All the sessions were conducted as a group activity by the principal investigator with session duration of 1 hour (40 minutes for education and 20 minutes for discussion).

After 8 weeks of education sessions, the women in the intervention group were invited to follow the given guidelines and they were followed up for a period of 6 months (August 2017–January 2018) by observing their adherence to the given guidelines. During this period, the women in the intervention group were addressed individually where necessary and family members' influence was modified accordingly. Estimation on current health status, cost effectiveness, feasibility, obtaining assistance, encouragement, or participation of closest family members or friends was also emphasized. They were asked to maintain a diary to include the activities they have done daily and daily diet which were prescribed to follow. The diary was evaluated at the progress meetings regularly and reminding phone calls were given frequently to enhance the compliance to follow-up.

The control group was not exposed to any planned education programme which allowed them to proceed with their usual lifestyle during this period, but we maintained contact with them regularly.

**Figure 1:**
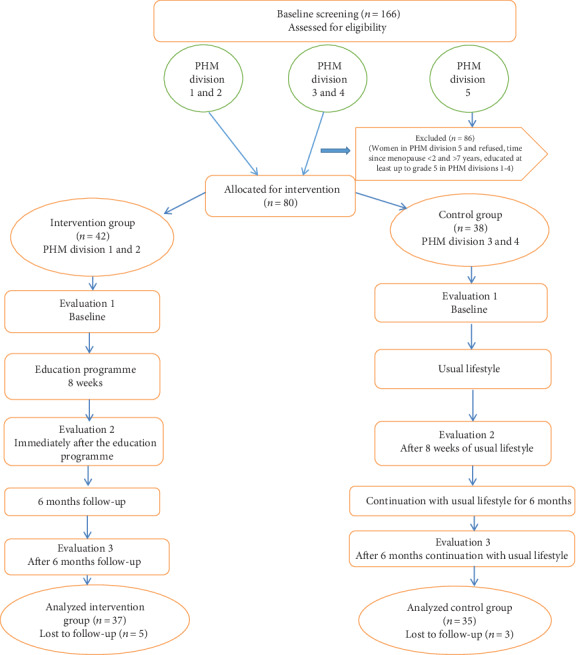
Flow diagram of the HPLEI (PHM area: public health midwifery area).

The study flowchart of the current is shown in Figure 1.

### 2.3. Evaluation of Knowledge, Attitude, and Quality of Life

Knowledge, attitude, MENQOL, and overall QOL were observed in both the intervention and control groups, separately at the baseline; Evaluation 1, after 8 weeks of baseline (immediately after the education programme of the intervention group); Evaluation 2, after the 6-month follow-up; Evaluation 3, by administering the knowledge and attitude questionnaire, menopause-specific quality of life (MENQOL) questionnaire, and Short-form 36 Survey (SF-36).

#### 2.3.1. Knowledge and Attitude Questionnaire

Knowledge on physiological basis of menopause, menopausal symptoms, complications of menopause, and health management after menopause was assessed in the knowledge section which contained 23 statements answered in a true/false/no idea manner. The attitude section included 13 statements for measuring the attitude which was designed as a five-choice Likert scale (strongly agree, agree, disagree, strongly disagree, and no idea). The knowledge and attitude statements were developed by the investigators using the statements which had been used to assess the knowledge and attitude of PMW in a previous study [21]. The reliability of the knowledge and attitude questionnaire has been evaluated locally (unpublished data).

In the knowledge section, every correct answer was given 1 mark; wrong answers and no idea were given 0 mark. Scores for four subscales and total score were created. The total score ranged from 0 to 23. In the attitude section, marks were given for each answer as strongly agree = 4, agree = 3, disagree = 2, strongly disagree = 1, and no idea = 0. Then, overall score was created, higher scores indicating higher level knowledge and positive attitude.

#### 2.3.2. MENQOL Questionnaire

This questionnaire has 29 items and is aimed at capturing MENQOL in areas centered on vasomotor, physical, psychosocial, and sexual functioning considered domains. It is a validated questionnaire internationally [22] and locally [23]. Higher scores indicate lower MENQOL.

#### 2.3.3. The SF-36 Survey

It is a multipurpose, short-form health survey consisting of 36 items, which provides a subjective estimation of the individual's QOL in eight domains under two main dimensions (physical and psychological). In SF-36, each dimension is given a score ranging from 0 to 100 using the original coding algorithm and higher values indicate higher QOL [24]. It has been validated internationally [24] and locally [25]. Higher scores indicate higher QOL.

### 2.4. Statistical Analyses

For the final analysis, only 72 women were included (intervention group = 37 and control group = 35). Descriptive data were presented as means and standard deviations (SD). The data gathered in all questionnaires were analyzed with the standard guidelines provided by the respective authors and publishers [22, 24].

The differences of basic characteristics between the intervention and control groups were compared using independent sample *t*-test. The follow-up data (Evaluation 3) of the intervention and control groups were analyzed using repeated measure ANOVA. *p* value was adjusted by the Bonferroni correction for multiple comparisons. The group^∗^time interaction and effect size was evaluated by assessing Wilk's lambda (*Λ*) and partial eta squared (*η*_p_^2^), respectively.

Furthermore, the difference between the variables obtained at the end of the 6-month follow-up (Evaluation 3) were further evaluated with one-way analysis of covariance (ANCOVA) while eliminating the effect of basic characteristics and baseline value of each variable.

### 2.5. Ethical Considerations

Ethical clearance for this study was obtained from the Ethics Review Committee, Faculty of Medicine, University of Ruhuna, Sri Lanka (Reference number; 31/05/2016:3.16). Informed written consent was obtained from all participants before the commencement of the study.

## 3. Results

### 3.1. Basic Characteristics of Participated Women

The mean (SD) age of the intervention and control groups was 54.6 (4.5) and 56.5 (3.4) years (*p* = 0.06), respectively. Age at menopause, time since menopause, and sociodemographic characteristics were not different between the intervention and control groups [18]. Knowledge, attitude, MENQOL, and overall QOL scores were not different between the intervention and control groups at the baseline (Evaluation 1) (*p* values are not shown in tables).

### 3.2. Changes of Knowledge, Attitude, MENQOL, and Overall QOL

Knowledge and attitude scores increased in the intervention group during the HPLEI (*p* < 0.001). In the control group, while a marginal increase in all dimensions of knowledge scores was seen, scores related to attitude remained unchanged. All scores of knowledge in the control group remained low when compared with the intervention group (Table 1).

All MENQOL scores decreased during the follow-up in the intervention group (p < 0.001) except the sexual domain (p = 0.32). However, scores increased in the control group (*p* < 0.001) with time (Table 2).

Except the social functioning and comfort domains, all other domains of QOL and the overall QOL scores increased (*p* < 0.001) in the intervention group during the study period. In the control group, except the vitality and comfort domains, all other domains and the overall QOL showed reduction (*p* < 0.05) (Table 3).

Between-group comparison at the end of the 6-month follow-up showed an improvement of knowledge (*p* < 0.05), attitude (*p* < 0.001) (Table 1), MENQOL except sexual domain (*p* < 0.001) (Table 2), and the overall QOL (*p* < 0.001) (Table 3) in the intervention group compared to the control group during the intervention.

The group^∗^time interaction was significant (*Λ* > 0.05, *p* < 0.001), and the main effect size was large revealed with moderate to large (*η*_p_^2^ > 0.2) for all the measured variables indicating the significant difference between the repeated measures over time in two groups (Tables 1–3).

Results did not change materially after controlling the effect of baseline characteristics with one-way ANCOVA (Tables 1–3).

## 4. Discussion

The current study revealed a positive impact of a HPLEI which was based on HPM on knowledge and attitude, and ultimately on enhancement of MENQOL and overall QOL in PMW. The current study supports the view that the importance of specific knowledge and attitude on menopause is needed to achieve the higher QOL.

Since we observed significant group^∗^time interaction, it is clear that the two groups changed over time, but in a different manner in all the variables studied. The moderate to larger effect size observed in all the variables emphasizes that there were significant differences between the groups over time.

Positive impact of education programmes focused on lifestyle management in improving different aspects of health status of PMW are seen consistently. Structured training programmes about menopause and its management have shown positive effects on knowledge [26–30] and positive attitudes towards menopause in PMW [30, 31]. Education programmes focused on lifestyle management have improved the MENQOL; vasomotor domain [19, 31], physical domain [16], psychosocial domain [31], and overall MENQOL [5, 32–34]. Significant improvement in sexual health has also been observed [35, 36] indicating that the sexual problems related to menopause are reversible. However, exercise awareness in PMW did not change the sexual QOL after 8 weeks of follow-up, while other aspects have improved [37]. Further, health education programmes on health improvement and maintenance of PMW have shown successful improvements in overall QOL in many domains [5, 19, 38–41]. Our previous publication also reported that HPLEI is an effective way to establish good living habits and general health status of PMW [18]. Therefore, study population, duration, and intensity of the programme would not be a matter if the programme showed beneficial and truthful effects as reported by many studies in similar nature [5, 13 26-41].

The effectiveness of an intervention depends on a careful, systematic design, guiding a useful framework and careful progress monitoring [42]. Therefore, in the individual focus, the success of our lifestyle education programme would be due to few reasons. It was a multifaceted, culturally accepted, well-designed programme guided by a theoretical framework. It also had the set realistic goals, individualized approach, and regular follow-up with strict monitoring in a lengthy period of 8 weeks training and 6 months follow-up [18].

The content validity of the education materials we developed were ensured by a group of experts, and the cultural acceptance was assured with another group of PMW. The content was not delivered as a whole, and it was a gradual process to emphasize the women to familiar and develop necessary skills gradually. Guiding the HPM, we were able to help the PMW to shape a positive behavior by focusing on the benefits, teach them to overcome obstacles to carrying out the behavior, and provoke high levels of efficacy and positive attitude through successful performance experience and positive feedback through regular follow-up [12].

Regular follow-up comprised of strict progress monitoring and motivating the women to engage the taught programme appropriately. Individual attention, group activities, involvement of family members, and providing adequate justifications and evidences on the effectiveness were also enhanced the positive outcomes.

The possible reason for having the improved QOL among the PMW in the intervention group would be due to the enhanced knowledge and understanding of women regarding the management of menopausal effects satisfactorily and positive attitude towards menopause. The other reason could be that they were able to get rid of irritable menopausal discomforts and enhanced general health status including physical functions, cardiovascular risks, and adiposity status [18] by following up the taught programme made their lives happier and healthier than earlier.

Therefore, this positive impact of the menopause-specific education programme based on lifestyle modifications encourages its use at the individual and community levels. The programme had multiple benefits including greater level of acceptability, affordability since it was inexpensive, and enhanced the awareness of women achieved through readily available options and remedies.

Therefore, health care professionals can utilize this information for health promotion in PMW at the individual and community levels by advising and informing about menopause to maintain health through health promotion strategies for possible problems. Information delivered with such attempt should be culturally accepted with affordable and available options to achieve realistic outcomes. It should further focus on positive aspects of menopause by supporting each individual woman's agenda. Therefore, we recommend establishing units in the community for educating PMW to promote healthy postmenopausal age with optimum physical and mental stamina for own health and well-being and for social activities.

Our study has a few strengths and limitations. We used an adequate number of matched samples for the study using a previous evidence of QOL improvement which was manageable to monitor adequately, minimized the contacts between the two groups with cluster randomization, and supervised their compliance strictly for a lengthy period of time. However, the self-reporting of variables with structured questionnaires could be considered a limitation. Therefore, we propose further studies with objective measurements of the variables or qualitative analysis of the sample for studied variables in the same area.

## 5. Conclusions

This study proved that education intervention focused on health-promoting lifestyle management was effective in improving knowledge, attitude, MENQOL, and overall QOL of PMW. This programme may offer implications for designing and implementing such interventions in future studies in this nature. This approach is recommended as a health care intervention in postmenopausal health management.

## Figures and Tables

**Table 1:** Comparison of mean scores of knowledge and attitude of intervention and control groups in three stages of evaluation (*n* = 72).

Parameter	Group	Evaluations			Within group comparison (*p* value)^a^	Between group comparison (*p* value)^a^	Group^∗^time interaction *Λ* (*p* value)	Effect size (*η*_p_^2^)	Between group comparison at the end of 6 months follow-up (*p* value)^b^
		E1 Mean (SD)	E2 Mean (SD)	E3 Mean (SD)					
*Knowledge*									
Knowledge on physiology of menopause	C	2.34 (0.83)	2.31 (0.79)	2.45 (0.70)	0.20	<0.001	0.37 (<0.001)	0.62	<0.001
	I	2.02 (0.83)	3.56 (0.50)	3.62 (0.49)	<0.001				
Knowledge on short-term effects of menopause	C	1.22 (1.00)	1.00 (1.22)	1.48 (1.59)	0.03	<0.001	0.09 (<0.001)	0.90	<0.001
	I	1.18 (1.07)	7.21 (0.71)	7.29 (0.70)	<0.001				
Knowledge on long-term effects of menopause	C	0.31 (0.67)	0.85 (1.06)	1.22 (1.05)	<0.001	<0.001	0.10 (<0.001)	0.89	<0.001
	I	0.32 (0.66)	5.89 (0.31)	5.91 (0.27)	<0.001				
Knowledge on management of menopause-related issues	C	0.57 (0.55)	2.05 (1.05)	4.54 (1.01)	<0.001	<0.001	0.11 (<0.001)	0.88	0.01
	I	0.27 (0.45)	4.83 (0.37)	4.86 (0.34)	<0.001				
Overall knowledge score	C	4.45 (1.44)	6.22 (1.84)	9.71 (2.21)	<0.001	<0.001	0.03 (<0.001)	0.97	<0.001
	I	3.81 (1.02)	21.51 (0.98)	21.70 (1.05)	<0.001				

*Attitude*									
Overall attitude score	C	24.42 (7.81)	24.74 (7.74)	23.91 (7.56)	0.58	<0.001	0.13 (<0.001)	0.86	<0.001
	I	24.05 (6.88)	27.35 (4.04)	44.02 (5.33)	<0.001				

E: evaluation; *Λ*: Wilk's lambda; *η*_*p*_^2^: partial eta squared. Groups: I: intervention; C: control. ^a^Means between and within the group were compared with two-way repeated measure ANOVA. ^b^Means between the groups at the end of 6 months were compared with one-way ANCOVA while controlling the baseline characteristics.

**Table 2:** Comparison of mean scores of MENQOL of intervention and control groups in three stages of evaluation (*n* = 72).

Menopause-specific quality of life	Group	Evaluations			Within group comparison (*p* value)^a^	Between group comparison (*p* value)^a^	Group^∗^time interaction *Λ* (*p* value)	Effect size (*η*_p_^2^)	Between-group comparison at the end of 6 months follow-up (*p* value)^b^
		E1 Mean (SD)	E2 Mean (SD)	E3 Mean (SD)					
Vasomotor domain score	C	9.45 (4.15)	10.28 (4.59)	12.80 (3.46)	<0.001	0.004	0.30 (<0.001)	0.69	<0.001
	I	8.62(2.83)	8.66(2.81)	7.89(3.49)	<0.001				
Psychosocial domain score	C	21.74 (7.21)	24.20 (8.03)	32.45 (7.40)	<0.001	0.001	0.10 (<0.001)	0.90	<0.001
	I	20.81 (7.72)	20.59 (7.42)	19.16 (8.19)	<0.001				
Physical domain score	C	60.48 (11.17)	67.57 (12.06)	83.34 (11.01)	<0.001	<0.001	0.05 (<0.001)	0.94	<0.001
	I	57.10 (14.49)	56.48 (14.65)	54.32 (16.23)	<0.001				
Sexual domain score	C	10.25 (4.61)	11.22 (4.38)	9.91 (3.02)	<0.001	0.64	0.65 (<0.001)	0.34	0.94
	I	11.10 (5.07)	11.00 (5.05)	10.67 (5.41)	0.32				
Overall menopause-specific QOL	C	101.94 (18.52)	113.28 (18.54)	138.51 (18.47)	<0.001	<0.001	0.04 (<0.001)	0.95	<0.001
	I	97.64 (25.94)	96.81 (25.80)	92.05 (28.87)	<0.001				

E: evaluation; *Λ*: Wilk's lambda; *η*_p_^2^: partial eta squared. Groups: I: intervention; C: control. ^a^Means between and within the group were compared with two-way repeated measure ANOVA. ^b^Means between the groups at the end of 6 months were compared with one-way ANCOVA while controlling the baseline characteristics.

**Table 3:** Comparison of mean scores of overall QOL of intervention and control groups in three stages of evaluation (*n* = 72).

QOL Scores	Group	Evaluations			Within group comparison (*p* value)^a^	Between group comparison (*p* value)^a^	Group^∗^time interaction *Λ* (*p* value)	Effect size (*η*_p_^2^)	Between group comparison at the end of 6 months follow-up (*p* value)^b^
		E1 Mean (SD)	E2 Mean (SD)	E3 Mean (SD)					
Physical functioning	C	68.80 (25.06)	68.91 (24.02)	54.94 (18.52)	<0.001	0.37	0.40 (<0.001)	0.59	<0.001
	I	64.89 (24.19)	65.00 (24.15)	76.35 (14.02)	<0.001				
Role performance due to physical problems	C	37.14 (43.45)	37.20 (42.51)	27.85 (32.59)	<0.001	0.01	0.57 (<0.001)	0.42	<0.001
	I	37.16 (44.33)	37.50 (43.72)	89.18 (17.22)	<0.001				
Role performance due to emotional problems	C	49.52 (46.70)	49.50 (46.64)	37.14 (35.02)	<0.001	0.02	0.70 (<0.001)	0.29	<0.001
	I	55.85(47.82)	56.71(47.19)	88.28(21.10)	<0.001				
Vitality (perception of energy or fatigue)	C	61.00 (21.37)	60.57 (20.92)	59.67 (19.99)	0.20	0.33	0.78 (<0.001)	0.21	<0.001
	I	63.78 (21.06)	63.70 (21.00)	67.43 (17.30)	0.001				
Social functioning	C	73.57 (22.02)	71.78 (21.07)	64.28 (14.89)	<0.001	0.97	0.59 (<0.001)	0.40	<0.001
	I	71.28 (23.54)	71.25 (22.51)	71.55 (23.05)	0.32				
Emotional well-being	C	75.88 (16.10)	75.65 (15.77)	69.94 (11.90)	<0.001	0.76	0.76 (<0.001)	0.29	<0.001
	I	73.29 (19.27)	73.40 (19.30)	75.13 (17.28)	0.01				
Comfort (perception of pain)	C	58.21 (23.98)	57.51 (22.11)	53.21 (18.38)	0.25	0.08	0.79 (<0.001)	0.20	<0.001
	I	64.52 (20.73)	63.81 (20.14)	66.28 (18.80)	0.16				
General health	C	56.00 (16.66)	55.57 (16.43)	41.24 (10.64)	<0.001	<0.001	0.27 (<0.001)	0.72	<0.001
	I	59.18 (17.77)	59.15 (16.22)	64.59 (15.38)	<0.001				
Physical health dimension	C	56.29 (22.14)	55.56 (19.47)	45.93 (15.16)	<0.001	0.01	0.34 (<0.001)	0.65	<0.001
	I	57.58 (21.59)	56.36 (19.31)	74.39 (9.24)	<0.001				
Psychological dimension	C	64.29 (20.95)	63.79 (20.47)	56.14 (14.50)	<0.001	0.07	0.57 (<0.001)	0.42	<0.001
	I	66.24 (21.55)	66.48 (21.50)	75.31 (13.71)	<0.001				
Overall QOL	C	60.29 (20.37)	59.65 (18.78)	51.03 (13.61)	<0.001	0.02	0.35 (<0.001)	0.64	<0.001
	I	61.91 (20.37)	61.42 (19.00)	74.85 (9.71)	<0.001				

E: evaluation; QOL: quality of life; *Λ*: Wilk's lambda; *η*_p_^2^: partial eta squared. Groups: I: intervention; C: control. ^a^Means between and within the group were compared with two-way repeated measure ANOVA. ^b^Means between the groups at the end of 6 months were compared with one-way ANCOVA while controlling the baseline characteristics.

## Data Availability

The data used to support the findings of this study are available from the corresponding author upon request.

## References

[1] Nutbeam D. (1998). Health Promotion Glossary. *Health Promotion International*.

[2] Waidyasekera H., Wijewardena K., Lindmark G., Naessen T. (2009). Menopausal symptoms and quality of life during the menopausal transition in Sri Lankan women. *Menopause*.

[3] Daly E., Gray A., Barlow D., McPherson K., Roche M., Vessey M. (1993). Measuring the impact of menopausal symptoms on quality of life. *BMJ*.

[4] The WHOQOL Group (1995). The World Health Organization quality of life assessment (WHOQOL): Position paper from the World Health Organization. *Social Science & Medicine*.

[5] Khajehei M., Moattari M., Mohit M., Rad M. S., Ghaem H., Forouhari S. (2010). The effect of education and awareness on the Quality-of-Life in postmenopausal women. *Indian Journal of Community Medicine*.

[6] Shakila P., Sridharan P., Thiyagarajan S. (2014). An assessment of women‘s awareness and symptoms in menopause (a study with reference to academic women‘s at Sri Lanka). *Journal of Business & Economic Policy*.

[7] Nazari M., Farmani S., Kaveh M. H., Ghaem H. (2016). The effectiveness of lifestyle educational program in health promoting behaviors and menopausal symptoms in 45-60-year-old women in Marvdasht, Iran. *Global Journal of Health Science*.

[8] Pérez-López F. R. (2004). An evaluation of the contents and quality of menopause information on the World Wide Web. *Maturitas*.

[9] Rice V. M. (2005). Strategies and issues for managing menopause-related symptoms in diverse populations: ethnic and racial diversity. *The American Journal of Medicine*.

[10] Galloway R. D. (2003). Health promotion: causes, beliefs and measurements. *Clinical Medicine & Research*.

[11] Pi-Sunyer F. X., Aronne L. J., Heshmati H. M., Devin J., Rosenstock J., Group R-NAS (2006). Effect of rimonabant, a cannabinoid-1 receptor blocker, on weight and cardiometabolic risk factors in overweight or obese patients: RIO-North America: a randomized controlled trial. *JAMA*.

[12] Pender N. J., Murdaugh C. L., Parsons M. A. (2006). *Health Promotion in Nursing Practice*.

[13] Yazdkhasti M., Simbar M., Abdi F. (2015). Empowerment and coping strategies in menopause women: a review. *Iranian Red Crescent Medical Journal*.

[14] Kalra B., Agarwal S., Magon S. (2012). Holistic care of menopause: understanding the framework. *Journal of mid-life health*.

[15] Cusack L., Del Mar C. B., Chalmers I., Gibson E., Hoffmann T. C. (2018). Educational interventions to improve people’s understanding of key concepts in assessing the effects of health interventions: a systematic review. *Systematic reviews*.

[16] Rotem M., Kushnir T., Levine R., Ehrenfeld M. (2005). A Psycho-Educational Program for Improving Women’s Attitudes and Coping With Menopause Symptoms. *Journal of Obstetric, Gynecologic, & Neonatal Nursing*.

[17] Rathnayake N., Lenora J., Alwis G., Lekamwasam S. (2019). Prevalence and severity of menopausal symptoms and the quality of life in middle-aged women: a study from Sri Lanka. *Nursing Research and Practice*.

[18] Rathnayake N., Alwis G., Lenora J., Lekamwasam S. (2019). Impact of Health-Promoting Lifestyle Education Intervention on Health- Promoting Behaviors and Health Status of Postmenopausal Women: A Quasi- Experimental Study from Sri Lanka. *BioMed Research International*.

[19] Moridi G. (2013). Quality of life among Iranian postmenopausal women participating in a health educational program. *Chronic Diseases Journal*.

[20] FAO (2001). Human energy requirements report of a joint FAO/WHO/UNU expert consultation. *Technical Report FAO Food and Nutrition Technical Report Series FAO*.

[21] Eslami A. A., Hassanzadeh A., Davari S., Noroozi E., Dolatabadi N. K. (2013). Knowledge and attitude toward menopause phenomenon among women aged 40–45 years. *Journal of education and health promotion*.

[22] Hilditch J. R., Lewis J., Peter A. (1996). A menopause-specific quality of life questionnaire: development and psychometric properties. *Maturitas*.

[23] Rathnayake N., Lenora J., Alwis G., Lekamwasam S. (2018). Cross cultural adaptation and analysis of psychometric properties of Sinhala version of Menopause Rating Scale. *Health and Quality of Life Outcomes*.

[24] Ware J. E. (2000). SF-36 health survey update. *Spine*.

[25] Gunawardena N. S., de Alwis Seneviratne R., Athauda T. (2006). Functional outcomes of unilateral lower limb amputee soldiers in two districts of Sri Lanka. *Military Medicine*.

[26] Hunter M., O’Dea I. (1999). An evaluation of a health education intervention for mid-aged women: five year follow-up of effects upon knowledge, impact of menopause and health. *Patient education and counseling*.

[27] Liao K., Hunter M. (1998). Preparation for menopause: prospective evaluation of a health education intervention for mid-aged women. *Maturitas*.

[28] Patel V., MSc Nursing Student, Sumandeep Nursing College, Sumandeep Vidyapeeth, Piparia, Vadodara-391760, Gujarat, India, Koshy S., Ravindra H. N. (2014). Effectiveness of structured teaching programme on knowledge regarding menopausal symptoms and its management among women. *IOSR Journal of Nursing and Health Science*.

[29] Kharat M., Kaur S. P. (2010). To assess the effectiveness of self instruction module on knowledge regarding menopausal changes and coping among pre-menopause women in selected areas of Wardha city. *International Journal of Nursing Education*.

[30] Taherpour M., Sefidi F. (2013). The effectiveness of education on the knowledge and attitude towards menopause symptoms and complications in postmenopausal women. *ZUMS Journal*.

[31] Booth-LaForce C., Thurston R. C., Taylor M. R. (2007). A pilot study of a Hatha yoga treatment for menopausal symptoms. *Maturitas*.

[32] Elavsky S., McAuley E. (2007). Physical activity and mental health outcomes during menopause: a randomized controlled trial. *Annals of Behavioral Medicine*.

[33] Shobeiri F., Jenabi E., Khatiban M., Hazavehei S. M. M., Roshanaei G. (2017). The effect of educational program on quality of life in menopausal women: a clinical trial. *Journal of menopausal medicine*.

[34] Yazdkhasti M., Keshavarz M., Khoei E. M. (2012). The effect of support group method on quality of life in post-menopausal women. *Iranian journal of public health*.

[35] Phillips N. A. (2000). Female sexual dysfunction: evaluation and treatment. *American Family Physician*.

[36] Osinowo H. O. (2003). Psychosocial factors associated with perceived psychological health, perception of menopause and sexual satisfaction in menopausal women and controls. *West African journal of medicine*.

[37] Ağıl A., Abıke F., Daşkapan A., Alaca R., Tüzün H. (2010). Short-term exercise approaches on menopausal symptoms, psychological health, and quality of life in postmenopausal women. *Obstetrics and gynecology international*.

[38] Farokhi F. (2015). Effect of skill life training in quality of life in menopausal women. *Scientific Journal of Hamadan Nursing & Midwifery Faculty*.

[39] Senba N., Matsuo H. (2010). Effect of a health education program on climacteric women. *Climacteric*.

[40] Ueda M., Matsuda M., Okano K., Suenaga H. (2009). Longitudinal study of a health education program for Japanese women in menopause. *Nursing & health sciences*.

[41] Sorpreso I. C. E. (2012). Health education intervention in early and late postmenopausal Brazilian women. *Climacteric*.

[42] Craig P., Dieppe P., Macintyre S., Michie S., Nazareth I., Petticrew M. (2008). Developing and evaluating complex interventions: the new Medical Research Council guidance. *BMJ*.

